# Pregnancy in women with advanced endometriosis and adenomyosis: possible complications and the role of surgery

**DOI:** 10.1186/s12958-025-01492-y

**Published:** 2025-11-26

**Authors:** Malin Brunes, Kristin Wennmo-Zuk, Hanna Åmark, Julia Wängberg Nordborg, Maria Forslund, Ligita Jokubkiene, Anna Marklund

**Affiliations:** 1 Department of Gynecology and Obstetrics, Södersjukhuset, Sjukhusbacken 10, Stockholm, 118 83 Sweden; 2 Department of Clinical Science and Education, Karolinska Institutet, Södersjukhuset, Stockholm, Sweden; 3 Department of Obstetrics and Gynecology, Institute of Clinical Sciences, Sahlgrenska Academy, University of Gothenburg, Gothenburg, Sweden; 4 Department of Gynecology and Obstetrics, Sahlgrenska University Hospital, Gothenburg, Sweden; 5 Department of Clinical Sciences Malmo, Obstetric, Gynecological and Prenatal Ultrasound Research, Lund University, Malmo, Sweden; 6 Department of Obstetrics and Gynecology, Skane University Hospital, Malmo, Sweden; 7 Department of Oncology-Pathology, Karolinska Institutet, Stockholm, Sweden; 8 Department of Reproductive Medicine, Division of Gynecology and Reproduction, Karolinska University Hospital, Stockholm, Sweden

**Keywords:** Endometriosis, Infertility, Surgery, Pregnancy, Live birth, Obstetric outcomes, Perinatal complications

## Abstract

**Introduction:**

Advanced endometriosis (stage III–IV, per the revised American Society for Reproductive Medicine (rARSM) classification of endometriosis) is associated with a range of pregnancy-related complications. Despite growing evidence, awareness of these risks remains limited among healthcare providers. Furthermore, the impact of endometriosis surgery prior to conception on pregnancy outcomes remains uncertain. The primary objective of this narrative review is to evaluate whether pre-conception surgery for advanced endometriosis influences early pregnancy, obstetric and neonatal outcomes. In the absence of robust surgical data, we also review outcomes in women with advanced endometriosis irrespective of surgical history, and we provide brief recommendations for pregnancy monitoring and delivery planning in this population.

**Findings:**

Evidence on how endometriosis surgery affects the risk of miscarriage is inconsistent, though most studies suggest a slightly increased risk in women with advanced disease, particularly in the presence of adenomyosis. For pre-eclampsia and hypertensive disorders, surgery does not appear to modify the risk and the higher rates seen in women with endometriosis may instead reflect co-excisting adenomyosis or the use of assisted reproductive technologies (ART). The risk of preterm birth is elevated in both surgically and conservatively managed groups, compared to the general population, but no significant difference is evident between the two groups. It remains uncertain whether endometriosis surgery or advanced disease itself increases the risk of delivering a small-for-gestational-age infant. Notably, foetal growth restriction appears to be more strongly associated with adenomyosis than with endometriosis. The risk of placenta praevia is reported to be increased in women with advanced endometriosis, but the impact of surgery remains unclear. Some studies suggest it may reduce the risk, while others report the opposite, possibly due to confounding by disease severity, adenomyosis or use of ART. Data on rarer outcomes—such as stillbirth, placental abruption, uterine rupture, haemoperitoneum, and bowel perforation—remain scarce.

Decidualised endometriomas can pose diagnostic challenges during pregnancy, though complications related to ovarian endometriosis in pregnancy appear rare. Finally, medical treatment of advanced endometriosis during pregnancy and the postpartum period is guided mainly by clinical experience and expert opinion, as high-quality evidence is lacking.

**Conclusion:**

While advanced endometriosis is linked to higher risks of pregnancy complications, there is no consistent evidence that pre-conception surgery reduces these risks. Well-designed, multicentre prospective studies are urgently needed to disentangle the roles of surgery, disease severity, adenomyosis, and ART. Meanwhile, individualised, multidisciplinary care remains essential, with careful documentation of endometriosis history and risk-stratified referral to tertiary centres when high-risk features are present.

## Background

Endometriosis has been linked to a variety of early pregnancy, obstetric and perinatal complications, as demonstrated in numerous studies and meta-analyses [1–4]. However, it is a heterogeneous disease with considerable variation in severity. While some patients have only small peritoneal lesions, others present with deep infiltrating disease, including complete obliteration of the pouch of Douglas and involvement of adjacent pelvic organs. Historically, the diagnosis and staging of endometriosis required surgical evaluation. Nowadays, advances in imaging techniques enable the diagnosis and staging using non-surgical methods such as magnetic resonance imaging (MRI) and ultrasound, as reflected in the 2022 European Society of Human Reproduction and Embryology (ESHRE) guidelines [5]. Helpful advancements in classification systems include the International Deep Endometriosis Analysis group (IDEA) protocol, the American Association of Gynecologic Laparoscopists (AAGL) classification, and the #ENZIAN classification [6–8].

While the association between severe endometriosis and adverse perinatal outcomes is known [9], the impact of surgical treatment on obstetric outcomes needs further research and clarification. A meta-analysis attempted to address this question in 2021, but it included only three studies [10]. Given the growing interest and body of evidence in this area, there is a clear need for a comprehensive narrative review.

This narrative review aims to summarise the current evidence on how surgical treatment prior to pregnancy in patients with revised American Society for Reproductive Medicine (rASRM) stage III-IV endometriosis (i.e., advanced endometriosis) affects early pregnancy, obstetric and neonatal outcomes. Given the limited data on the direct role of surgery, we also review how advanced endometriosis itself may influence these outcomes and consider potential confounders such as disease severity, co-existing adenomyosis, and the use of assisted reproductive technologies (ART). Drawing on both the reported evidence and the clinical experience of the authors, we provide recommendations for pregnancy monitoring and delivery planning in this population.

## Method

This article is based on a critical review of the peer-reviewed indexing US National Library of Medicine’s PubMed database. A search strategy was developed together with a librarian, and included the following search terms: ((endometrioma* OR endometriosis OR adenomyosis) pregnan* (surgery OR surgical OR postsurg* OR postop*)) AND (preterm OR small for gestational age or growth restriction OR hypertension OR pre-eclampsia OR cholestasis OR caesarean OR haemorrhage OR placenta praevia OR placental abruption OR prematur* OR miscarriage OR implantation rate OR live birth rate OR clinical pregnancy rate OR mid-trimester loss OR cycle cancellation OR ovarian response OR reproductive outcome OR obstetric outcome OR pregnancy outcome) AND (2017:2025[pdat]). The language was limited to English. The publication year was limited to 2017 onward, as this narrative review focuses on recent advancements in the field.

**Fig. 1:**
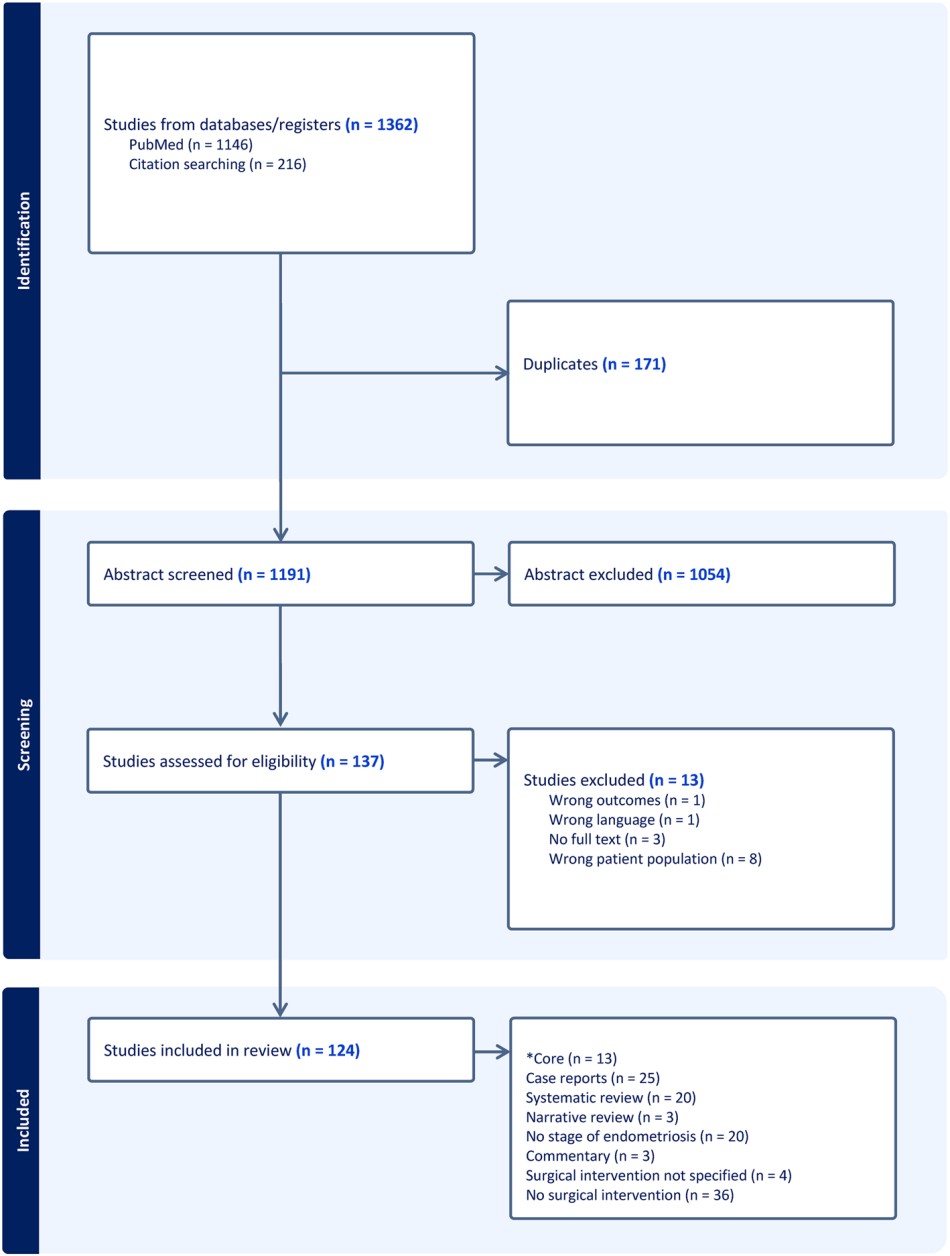
*Core = PICO framework (Population, Intervention, Control, Outcomes). Thirteen studies reported pregnancy complications and obstetric outcomes (O) in women with stage III–IV endometriosis (P) who had undergone surgical intervention (I), compared with women managed conservatively or women without endometriosis (C). Benaglia et al. [11], Farella et al. [12], Lapointe et al. [13], Miura et al. [14], Nirgianakis et al. [15], Ono et al. [16], Porpora et al. [17], Salamun et al. [18], Schliep et al. [19], Thomin et al. [20], Tuominen et al. [21], Uchida et al. [22], Mooney et al. [10]

The searches were performed between 14 February and 20 March, 2025. All studies were exported to Covidence for screening. Duplicate papers were excluded. Abstract and full-text screening were conducted independently by two researchers, and any disagreements were discussed and resolved within the research team (Fig. 1).

Randomised controlled trials (RCTs), systematic reviews and meta-analyses were prioritised, followed by cohort studies. Descriptive studies and case reports were considered for uncommon outcomes. For some outcomes, no studies comparing surgery versus no surgery could be found and the risk of these complications among women with advanced endometriosis was described irrespective of previous surgery.

No data extraction was performed. No ethical approval was required, as this narrative review utilises data from previously published studies. We used ChatGPT (OpenAI) to assist with grammar, and spelling checks during the preparation of this text.

## Results

The search yielded 1362 records, of which 171 duplicates were removed. After title and abstract screening (1191 records), 137 full texts were assessed for eligibility, and 124 studies met inclusion criteria (Fig. 1). No RCTs were identified. Data extraction and synthesis were structured according to the PICO framework (Population, Intervention, Control, Outcomes). Thirteen studies reported pregnancy complications and obstetric outcomes (Outcomes) in women with stage III–IV endometriosis (Population) who had undergone surgical intervention (Intervention), compared with women managed conservatively or women without endometriosis (Control).

### Risk of miscarriage and ectopic pregnancy

The ESHRE states that the available data on miscarriage rates in women with endometriosis compared to those without endometriosis are somewhat conflicting, although most studies and systematic reviews observe an increased risk [5]. Part of the variability in reported miscarriage risk may stem from varying definitions of miscarriage regarding gestational weeks, and inclusion of different stages of endometriosis within the same study.

Only one study identified in our search specifically examined the risk of miscarriage in relation to previous endometriosis surgery [16]. This Japanese retrospective cohort study included 208 pregnant women with a history of severe endometriosis (r-ASRM stage IV). The risk of miscarriage tended to be lower in the surgery group than in the non‐surgery group (12.2%, 18/148 vs. 21.7%, 13/60, respectively; *p* = 0.089), but the difference was not statistically significant [16].

In a meta-analysis and systematic review from 2024, the risk of miscarriage in women who had undergone surgery for endometriosis prior to conception was higher than in women without endometriosis (odds ratio (OR) 1.77, 95% confidence interval (CI) 1.20—2.62) [23]. Increased risk of miscarriage was found for deep endometriosis (DE) (OR 1.51, 95% CI 1.18—1.92), but not for ovarian endometrioma (OR 1.20, 95% CI 0.98—1.47). Notably, endometriosis per se was not shown to increase the risk of miscarriage in non-ART pregnancies. For ART pregnancies, the association between endometriosis and miscarriage was shown in the analysis of pooled data (33 studies, OR 1.24, 95% CI 1.08—1.42), but not in the analysis adjusted for maternal age (10 studies, OR 1.24, 95% CI 1.00—1.53). However, adenomyosis was shown to be associated with miscarriage both in the main analysis and in the sub-analysis of ART-pregnancies [23]. Similarly, a meta-analysis from 2020 found an increased risk of miscarriage in spontaneous pregnancies among women with endometriosis (OR 1.81, 95% CI 1.44—2.28), but not in ART pregnancies (OR 1.03, 95% CI 0.92—1.14), when compared to women undergoing ART due to tubal factor infertility. Women with adenomyosis had a higher miscarriage risk also after ART (OR 2.81, 95% CI 1.44—5.47) [24].

Ectopic pregnancy is another potential life-threatening complication of early pregnancy. A meta-analysis from 2020 reported a more than two-fold risk of ectopic pregnancy in women with endometriosis without differentiation according to the stage of endometriosis or if the women were pregnant spontaneously or after ART [25].

Clinicians should be aware of the possible increased risk of miscarriage and ectopic pregnancy in women with endometriosis. Additionally, future research should focus on large studies, particularly according to the stage of endometriosis (including adenomyosis), the role of surgery prior to conception, and the mode of conception [5].

### Obstetric and perinatal outcomes

Endometriosis has been linked to an increased risk of perinatal complications [18, 13, 4], potentially due to a chronic inflammatory state, affected angiogenic signaling and impaired spiral artery remodeling [4, 9]. These factors may interfere with implantation and placentation, thereby impacting pregnancy progression and late pregnancy outcomes [4, 9]. The crucial question is whether surgical treatment of advanced endometriosis can modify these mechanisms and improve perinatal outcomes.

#### Pre-eclampsia and pregnancy hypertension

None of the included studies reported any significant difference in the incidence of hypertension or pre-eclampsia when comparing surgical treatment for endometriosis with conservative treatment [15, 16, 21, 22]. According to Berlac et al., women with endometriosis had an increased risk of pre-eclampsia (OR 1.4, 95% CI 1.3—1.5) and of severe hypertensive complications such as severe pre-eclampsia, eclampsia, or Haemolysis, Elevated Liver enzymes, Low Platelet count (HELLP) syndrome (OR 1.7, 95% CI 1.5—2.0) compared to women without endometriosis [26]. However, a key limitation of the study was the lack of data on endometriosis stage and potential co-existing adenomyosis. A 2024 systematic review and meta-analysis did not find a specific association between stage III–IV endometriosis and pre-eclampsia, but instead reported increased risk linked to adenomyosis (OR 1.70, 95% CI 1.16—2.48) and to ART-related factors, such as artificial-cycle frozen embryo transfer (OR 2.14, 95% CI 1.91—2.39) [23].

#### Preterm birth

Farella et al. reported a higher prevalence of preterm birth in women with a history of surgical management for endometriosis, compared with the French general population [12]. Identified independent risk factors included ART, body mass index (BMI) > 30, endometriosis infiltrating the bladder, and rectal surgery [12].

Some other studies compared the prevalence of preterm birth in women with surgical versus conservative treatment of endometriosis and found no clinically relevant difference between the two groups [14, 16, 19–21]. However, both groups had a higher incidence of preterm birth compared to the general population [21]. Similarly, a meta-analysis from 2024 found endometriosis rASRM stage III-IV to be a considerable risk factor for preterm birth (OR 1.43, 95% CI 1.32 −1.56), regardless of the method of conception [23].

#### Small for Gestational Age (SGA)

In a retrospective cohort study by Ono et al. including 208 pregnant women with a history of endometriosis, no significant difference in the proportion of SGA infants was observed between surgical and conservatively treated groups, while a tendency to lower incidence of foetal growth restriction in the surgical group was reported [16]. However, a French study from 2020 reported an increased prevalence of SGA in women with a history of surgical management of endometriosis when compared to the general population [12]. An important limitation of these studies was the lack of data on co-existing adenomyosis. In a meta-analysis from 2019, adenomyosis but not endometriosis was associated with increased risk of SGA [4].

#### Stillbirth

The studies included in this narrative review generally lacked sufficient population size to detect differences in rare outcomes such as stillbirth. Thus, findings should be interpreted with caution. Two studies reported on stillbirth but found no significant differences between the group with surgical and conservative treatment [19, 21].

#### Antepartum and postpartum haemorrhage

No studies compared the risk of antepartum or postpartum haemorrhage after surgery for advanced endometriosis compared with conservative treatment*.* However, a higher incidence of antepartum haemorrhage has been shown among pregnant women with endometriosis compared with pregnant women without endometriosis [26].

#### Placenta praevia

In a meta-analysis from 2024, Busnelli et al. found that women with stage III–IV endometriosis had a significantly increased risk of placenta praevia compared to controls (OR 6.61, 95% CI 2.08—20.98), and the risk was even higher in those with DE (OR 14.54, 95% CI 3.67—57.67) [23]. In contrast, no significant association was found between ovarian endometrioma and placenta praevia. Women who underwent pre-pregnancy surgery for endometriosis had a significantly higher risk of placenta praevia compared to women without endometriosis (OR 7.90, 95% CI 2.80—22.26). Additionally, surgical treatment of endometriosis was associated with a higher risk of placenta praevia than conservative treatment (OR 2.05, 95% CI 1.67—2.53). Similarly, in a case–control study by Nirgianakis et al., women who had undergone laparoscopic excision of posterior DE were matched with controls with no history of endometriosis, and a higher risk of placenta praevia was observed in the pregnancies after surgery for posterior DE [15].

In contrast, Ono et al. found a lower prevalence of placenta praevia in women with previous surgical treatment of endometriosis as compared to conservative treatment group (8.5% vs. 23.4%, *p* = 0.02). Placenta praevia was more prevalent in pregnancies that occurred later than two years than within two years after surgery (20% and 2.4%, respectively, *p* = 0.002) [16]. Similarly, Uchida et al. reported that among patients with r-ASRM endometriosis stage III-IV, the incidence of placenta praevia was significantly lower in pregnancies with prior complete surgical treatment compared to those with previous conservative treatment (5.9% versus 33.3%, *p* = 0.038) [22].

In a study by Farella et al., a higher rate of placenta praevia was found in women with a history of surgical treatment of endometriosis, compared with the general population (1.7% and 1.1%, respectively) [12]. Conception with the use of ART was identified as an independent risk factor related to placenta praevia. However, previous studies reporting an increased risk of placenta praevia in women with endometriosis found that the risk remained higher even after controlling for ART [26, 27].

Taken together, the heterogeneous findings across surgical and non-surgical cohorts likely reflect confounding by disease severity, adenomyosis, and ART. At present, there is no robust evidence that pre-conception surgery reduces the elevated placenta praevia risk observed in advanced disease.

#### Placental abruption and uterine rupture

Several studies have reported an increased risk of placental abruption as well as of uterine rupture among women with endometriosis [3, 16]. However, no studies were found comparing pregnancy outcomes between women with endometriosis who underwent surgical treatment and those who did not.

#### Haemoperitoneum

Spontaneous haemoperitoneum during pregnancy is rare, with an estimated incidence of 0.4–0.5/10 000 births [28, 29]. In the context of endometriosis, haemoperitoneum may theoretically originate from a ruptured endometrioma, bleeding from the utero-ovarian vessels, or erosion of pelvic blood vessels by endometriotic implants. To evaluate the magnitude of risk of spontaneous haemoperitoneum among ART pregnancies, Benaglia et al. conducted a retrospective case series including 362 pregnancies in 348 women with a history of surgery for endometriosis, including ovarian endometriomas or deep endometriotic nodules detected by ultrasound before ART [11]. One case of spontaneous haemoperitoneum was recorded (in a woman without previous surgery for endometriosis), corresponding to a rate of 0.3%.

#### Bowel perforation

Bowel perforation is a rare but potentially life-threatening complication during pregnancy among women with severe endometriosis [30]. However, no studies were found comparing outcomes in those with and without prior surgical treatment.

#### Mode of delivery

A cohort study by Thomin et al., including 72 pregnancies in 67 women followed for colorectal endometriosis, explored delivery methods and complications in those with (41 women) and without (26 women) previous colorectal surgery for endometriosis [20]. Caesarean delivery occurred in half of the women and was associated with a high rate of postoperative complications (39%) compared with vaginal delivery (14%). However, no significant difference was found between those with versus without previous surgical treatment of endometriosis. Complications—such as endometritis, wound infection, postpartum haemorrhage, and anemia—occurred in 67% of women with anterior deep infiltrating endometriosis, compared to 26% of those without the condition, regardless of prior surgical history [20].

A meta-analysis by Busnelli et al. found that women who underwent pre-pregnancy surgery for endometriosis had a significantly higher risk of cesarean section compared to women without endometriosis (OR 1.71, 95% CI 1.27—2.31. Additionally, one of the studies included in this meta-analysis showed a higher cesarean section rate in women who had surgery for endometriosis compared to those with untreated endometriosis (OR 1.72, 95% CI 1.59—1.86) [26]. However, no significant association between stage III/IV endometriosis and cesarean section was found, either in crude (OR 0.82, 95% CI 0.27—2.50) or age-adjusted analyses (OR 0.49, 95% CI 0.16—1.5), though heterogeneity was high. In contrast, ovarian endometriosis was associated with an increased risk of cesarean delivery (OR 1.67, 95% CI 1.16—2.40), but this association was not confirmed after adjusting for maternal age in one study. Women with DE showed a significantly higher risk of cesarean section, both in unadjusted (OR 2.34; 95% CI: 1.71–3.19) and adjusted analyses (OR 2.20; 95% CI: 1.38–3.51), with low to moderate heterogeneity.

In summary, most studies indicate an increased risk of cesarean delivery in women with advanced endometriosis. However, the specific impact of prior endometriosis surgery on delivery mode and associated complications remains uncertain.

#### Antenatal care and delivery

Women with advanced endometriosis are at increased risk for rare but serious perinatal complications [9]. If a patient has advanced disease and/or a history of surgical treatment, this information should be clearly communicated to the midwives and physicians involved in antenatal care and labour. Awareness of these risk factors may facilitate timely recognition and management of potentially life-threatening complications associated with DE.

When no clear medical indication for cesarean section exists, vaginal delivery should be the preferred option. If cesarean section is necessary, it is important to consider the nature and complexity of any previous surgical treatment. In cases involving extensive prior surgery, particular caution is warranted, and surgical expertise should be ensured. During caesarean section, the surgeon should assess for dense posterior uterine adhesions before attempting uterine exteriorisation. If such adhesions are present, intra-abdominal closure of the uterotomy is recommended unless extensive and complete adhesiolysis has first been performed.

Deep infiltrating endometriosis has been implicated in rare but severe complications such as spontaneous haemoperitoneum in pregnancy. However, given the rarity of these events and the absence of robust evidence that pre-conception surgery reduces obstetric risk, routine surgical treatment prior to conception cannot be recommended solely for the purpose of preventing pregnancy complications. Current data do not support a reduction in early preterm birth or placenta praevia after pre-conception surgery, the latter being the most consistently observed complication in advanced disease. Surgery before pregnancy should therefore be reserved for symptom relief or fertility indications, rather than as prophylaxis against obstetric morbidity.

From a practical perspective, a risk-stratified approach is advisable: women with mild disease and no high-risk features can usually be managed within standard obstetric services, whereas those with severe endometriosis, extensive prior pelvic surgery, or suspected placenta praevia/accreta should be systematically referred to tertiary centres with access to experienced obstetricians, colorectal/urological surgeons, interventional radiology, and blood bank support.

### Adnexal complications during pregnancy

Adnexal complications during pregnancy are rare, however, they may present diagnostic and management challenges.

Endometriomas are found in 0.2–2.5% of women during the first trimester of pregnancy at routine ultrasound examination and in women with early pregnancy complications [31–33]. As many as 75% of endometriomas may undergo changes during pregnancy [34]. Decidualisation is the most common finding reported in 12–58% of women [31, 34–38]. Decidualisation is believed to occur under the influence of progesterone leading to transformation of endometrial stromal cells in the ectopic endometrium. It can be detected by ultrasound and appears as a presence of solid components or rounded papillary projections with smooth surface, often moderately or richly vascularised [38–40]. Decidualisation can be detected as early as 11–13 gestational weeks [41], with a median time of 17 weeks [34]. Detection time also depends on the timing of ultrasound examination and when the endometrioma is detected. Therefore, incorporating detailed ovarian assessment in the first-trimester ultrasound may be of benefit.

Because of solid components and papillary projections, morphological appearance of decidualised endometriomas mimics malignancy and can lead to misdiagnoses and subsequent unnecessary surgery. Documented benign endometrioma before the pregnancy or concomitant DE at ultrasound examination during pregnancy, might increase probability of the cyst with papillary projections being decidualised endometrioma rather than borderline tumour, however, the diagnosis might require ultrasound expertise. Assessment of the surface of the papillary projections might help in differentiating decidualised endometriomas from serous borderline ovarian tumours: smooth surface of papillary projections is typical for decidualised endometriomas while papillary projections with irregular surface is a sign of borderline malignancy [38, 42].

During pregnancy, most endometriomas decrease in size and may completely resolve [34, 36, 43], which reflects natural behaviour of endometriomas. In some endometriomas initial enlargement and thereafter regression may be observed [34, 36]. It is believed that only endometriomas covered with the endometrium may undergo decidualisation [21, 25]. While size of endometriomas tend to decrease during pregnancy, size of solid components in the decidualised endometriomas seems to increase [34]. Decidualisation signs regress within 8 weeks after delivery [34].

Although endometriomas during pregnancy are rare, it is important that clinicians are aware of the potential for decidualisation, as it may impact further management of the patients. Complications related to endometriomas, such as cyst rupture or ovarian torsion, are rare in both pregnant and non-pregnant women [37, 44, 45]. In a multicentre prospective long-term follow-up study, ovarian torsion has been reported in 0.4% and cyst rupture in 0.2% of surgically removed cysts in women for 24 months follow-up [45]. None of the ovarian masses with torsion were endometriomas, while 50% of the cysts with rupture were confirmed being endometriomas. Endometriomas are less likely to undergo torsion due to the presence of adhesions that typically restrict their mobility. Out of 315 adnexal masses with confirmed torsion at surgery, only one (0.6%) was endometrioma [44].

There is little known on the cyst-related complications during pregnancy. In a recent retrospective study, cysts-related complications, such as torsion and cyst rupture, during pregnancy occurred in 1.5% of women, with none of the affected cysts identified as endometriomas [7]. Infected endometriomas is a rare event, described as isolated case reports rather than large-scale follow-up studies. Infection of an endometrioma may be associated with oocyte retrieval during assisted reproductive treatments, particularly when the cyst is punctured, although such infections can also arise spontaneously without prior intervention [46, 47]. Women with advanced endometriosis might be at an increased risk of infected endometriomas as well [47].

To the best of our knowledge, there are no studies describing infected endometriomas during pregnancy, which may depend on the generally low prevalence of endometriomas during pregnancy.

In pregnancies with adnexal masses of presumed endometriotic origin, conservative management is generally appropriate because most endometriomas regress or remain stable. Surgery is reserved for rapid growth, suspicion of malignancy, or acute complications (torsion/rupture). When intervention is required, it should be undertaken in a tertiary centre with obstetric and gynaecological surgical expertise. At present, there is no clear evidence supporting routine surgical treatment of endometriosis prior to pregnancy in order to reduce the risk of obstetric complications.

### Hormonal therapy and pain management during and after pregnancy

In women with advanced (rASRM stage III–IV) endometriosis, little is known about the natural history regarding pain and endometriosis during pregnancy, regardless of whether surgical treatment has been performed prior to conception [48]. Current knowledge is largely based on case reports or very small cohorts, lacking data on the temporal development of pain [49]. Historically, pregnancy was thought to have a “curative” effect on endometriosis, with symptom relief first described as early as 1953 [50]. More recent data suggest that many women do experience improvement, most likely due to progesterone dominance, pregnancy-induced amenorrhea, and immunological changes that may suppress lesion activity and the development of new implants [51–55].

From a surgical perspective, the influence of pre-pregnancy excision of advanced disease on pain trajectories during pregnancy has not been adequately studied. While surgery may reduce baseline symptom burden before conception, it remains unclear whether this translates into different pain experiences or complications during pregnancy compared to women with untreated disease. Given the infiltrative nature of stage III–IV lesions and frequent coexistence of adenomyosis, residual pain symptoms may persist even after surgery, and symptom relief during pregnancy may therefore be heterogeneous [17, 48, 49].

Currently, no guidelines provide specific recommendations on pain management in pregnant women with a history of endometriosis surgery or with untreated advanced disease [5, 56, 57]. Management should follow general obstetrical principles, noting that opioids are transferred to the foetus and can affect the newborn child.

Postpartum, the timing of hormonal therapy resumption is another unresolved issue, particularly in surgically treated patients where recurrence prevention is a major concern. Although evidence is lacking, it is generally believed that hormonal treatment should start early, ideally with pre-delivery planning. While estrogen-containing substances should be avoided at least the first month after delivery due to the elevated risk for deep venous thrombosis, progestins have no such effects. This approach is particularly relevant for women with surgically treated stage III–IV disease, where long-term recurrence risk and residual lesions remain important clinical considerations.

## Conclusions

Endometriosis is increasingly recognised not only as a chronic gynaecological condition with implications for fertility but also as a potential risk factor for adverse perinatal outcomes. Women with advanced endometriosis appear to face higher rates of complications during pregnancy, including miscarriage, preterm birth, placenta praevia, hypertensive disorders, and an increased likelihood of caesarean section. [4, 23, 58, 59]. Rare but potentially life-threatening complications such as haemoperitoneum are also reported in this population. Spontaneous haemoperitoneum is thought to result from bleeding of endometriotic implants or fragile utero-ovarian vessels in the context of advanced disease, but despite its dramatic presentation the event remains exceedingly rare, and no evidence exists that pre-conception surgery prevents its occurrence [11, 28, 29].

Management of decidualised endometriomas may present a clinical challenge, as their appearance on ultrasound may mimic malignancy. Referral for a second-opinion ultrasound expert is recommended before deciding on further management. Medical treatment of endometriosis and endometriosis-related pain during and immediately after pregnancy is currently based mainly on clinical experience, and there is an obvious need for further research in this field.

Surgical excision of endometriotic lesions is often pursued to alleviate pain or improve fertility outcomes. Yet, the extent to which such intervention influences pregnancy and birth outcomes remains unclear. While some hypothesise that surgery prior to conception may reduce inflammation [60], improving uterine and placental function, others raise concerns that it could contribute to tissue scarring, thereby introducing new risks [12].

Currently, there is a lack of high-quality, prospective data comparing pregnancy outcomes in women with endometriosis who have undergone surgery prior to pregnancy versus those who have not. Retrospective studies and registry-based analyses are limited by selection bias and heterogeneity in disease staging, surgical techniques, and timing relative to conception. From the available literature, it is impossible to ascertain whether results from the comparisons between surgical and conventional treatment are biased by the exact stage and type of endometriosis [30]. Moreover, accumulating evidence suggests that several obstetric complications attributed to advanced endometriosis may be driven by co-existing adenomyosis. This distinction helps explain why excisional surgery for pelvic endometriosis has not consistently reduced risks such as miscarriage, pre-eclampsia, or placenta praevia. Detrimental effects of co-existing adenomyosis and use of ART in the context of surgical versus non-surgical pre-conceptional management of advanced endometriosis have so far been difficult to disentangle [9, 10, 12, 23]. Therefore, there is a recognised need for well-designed RCTs to determine whether surgical management of endometriosis prior to conception exerts a protective, neutral, or detrimental effect on pregnancy outcomes. In parallel, prospective cohort studies—ideally involving collaborative efforts to achieve sufficient statistical power—should be encouraged to account for potential confounders such as ART and co-existing adenomyosis. Together, these studies are essential to advancing our understanding of the relationship between endometriosis, its surgical management, and pregnancy outcomes.

In the meantime, clinical decision-making should remain individualised. Given current evidence and the frequent co-occurrence of adenomyosis, pre-conception surgery for deep endometriosis should not be recommended solely to improve obstetric outcomes. Surgical management retains a role for pain relief and fertility optimisation, but its contribution to safer pregnancies remains unproven. High-risk cases (e.g., suspected placenta praevia/accreta) should be planned for delivery in tertiary centrs. Multidisciplinary management involving reproductive specialists, maternal-foetal medicine, and endometriosis experts is essential to tailoring care and counselling. The data and collective clinical experience generated through this approach should also serve as a valuable foundation for future research in the field.

## Data Availability

No datasets were generated or analysed during the current study.

## Abbreviations

AAGL: American Association of Gynecologic Laparoscopists
ASRM: American Society for Reproductive Medicine
ART: Assisted reproductive technologies
CI: Confidence interval
DE: Deep endometriosis
ESHRE: European Society of Human Reproductive and Embryology
HELLP: Hemolysis, Elevated Liver enzymes, Low Platelet count
IDEA: International deep endometriosis analysis
MRI: Magnetic resonance imaging
OR: Odds Ratio
RCTs: Randomized controlled trials
SGA: Small for Gestational Age
